# Explicit Sense of Agency in an Automatic Control Situation: Effects of Goal-Directed Action and the Gradual Emergence of Outcome

**DOI:** 10.3389/fpsyg.2020.02062

**Published:** 2020-08-31

**Authors:** Ryoichi Nakashima, Takatsune Kumada

**Affiliations:** ^1^Department of Psychology, The University of Tokyo, Bunkyo, Japan; ^2^RIKEN CBS-TOYOTA Collaboration Center, RIKEN, Wako, Japan; ^3^Graduate School of Informatics, Kyoto University, Kyoto, Japan

**Keywords:** sense of agency, automatic control, manual control, goal-directed action, gradual effect

## Abstract

Sense of agency (SoA), or the subjective feeling that “I am the agent controlling the object,” is essential for learning and enjoying object manipulation. Recently developed automatic control systems, such as the cruise control systems in autonomous vehicles, require less manual control from the manipulators. It has to date been impossible to completely relieve operators of the need for manual control in many automatic control systems developed for tool-using situations. Therefore, it is important to examine how to maintain SoA (illusorily) during an automatic control situation. We investigated the effects of two typical characteristics of everyday tool-use situations on SoA when braking a moving object with a keypress. These characteristics included the presence of a goal (e.g., in driving situations, the driver steps on the brake pedal to stop the car at an expected position) and the gradual emergence of the outcome (e.g., the driver steps on the brake pedal and the car usually slows down first and then stops). We conducted an experiment in which participants stopped a moving object and then rated their SoA for stopping the object. Participants were explicitly informed that the object would sometimes stop independently of their keypress. Results showed that both characteristics decreased SoA in the manual control situation but increased SoA in the automatic control situation. Thus, these characteristics could be useful for maintaining a sense of agency in automatic control situations.

## Introduction

As people interact with their environment, their knowledge of how their own actions affect the environment often manifests itself as “sense of agency” (SoA) or the subjective feeling of controlling external events by means of their own actions (Haggard and Tsakiris, 2009; Haggard and Chambon, 2012). Nowadays, people frequently use tools to control external events and perform many tasks, such as driving a car and manipulating computers. When people use tools, they usually have the subjective feeling that “I am controlling this tool.” This study refers to this feeling as SoA for controlling objects.

In the traditional explanation of SoA (e.g., Blakemore et al., 1998, 1999; Voss et al., 2006), a predicted state is generated from an efferent copy of one’s motor command and then compared with the actual state of the controlled object. If these states match, SoA is felt; if they do not match, SoA is not produced (e.g., Franck et al., 2001; Farrer et al., 2008). However, the underlying mechanism may not be so straightforward. For example, external cues or inferences can generate an “illusory” SoA (Wegner et al., 2004; Moore et al., 2009). Recent theory suggests that the judgment of agency may be a postdictive or reconstructive process (Haggard, 2005; Metcalfe and Greene, 2007; Wen et al., 2015a,b), based on an explicit inference or interpretation and/or an implicit comparison between expected and actual perceptual information.

Although tool manipulation usually results in SoA, the recent development of automatic control systems, which require less manual control on the operator’s part, should inhibit this feeling. One of the best-known examples of an automatic control system is a cruise control system, which many automotive manufacturers have begun to include in autonomous vehicles. When people drive an autonomous car, they feel that driving is easier because they no longer need to control the car by themselves. At the same time, they may feel less engaged in the driving task because they lack SoA. Indeed, a previous study (Berberian et al., 2012a) of SoA in aircraft automation systems demonstrated that when the level of automation of a supervision task was increased from full manual control to full automatic control, SoA decreased.

What problems arise when a tool works automatically? Developers of automation systems have often believed that adding automation is a simple substitution of a machine activity for human activity and that automation produces a reduction in SoA because of this substitution (cf. Berberian et al., 2012b). However, traditional automation has many negative consequences that arise from human “out-of-the-loop” performance (Endsley and Kiris, 1995; Kaber and Endsley, 1997), which impairs the ability of operators in an automatic system to take over manual operations in the event of automation failure. At present, in an automatic control situation, operators may sometimes need to take action for control because the system cannot deal with all the problems possibly arising. It is especially difficult for the system to handle dangerous and complex problems, and operators are required to take over control in an emergency. In these situations, the “out-of-the-loop” performance problem has potentially serious consequences.

It is obviously necessary for the operator to be involved in the control for preventing this “out-of-the-loop” performance problem. An effective way to accomplish this is to maintain SoA during tool-use or object control (see Berberian et al., 2012b; Wen et al., 2019), although there may be other solutions. If operators explicitly feel SoA while controlling tools, they should also feel that they are involved in the tool-use loop. That is, feeling SoA should be the basis for feeling involvement in the tool-use loop even in an automation system. We examined whether it is possible to maintain SoA while moving an object during an automatic control situation. It should be noted that SoA during an automatic control situation is, strictly speaking, an illusory SoA because observers do not control the object at all. Therefore, in order to maintain SoA during an automatic control situation, observers should feel like they are controlling the object.

The present study focused on two possible characteristics of the object control situation that influence SoA during an automatic control situation. The first characteristic is goal-directed action. In many tool-use situations, operators use the tool to achieve their goal (i.e., to carry out their intention). Consider a situation when a driver brakes a car by stepping on the brake pedal to stop the car at an expected position (e.g., at a stop sign or in a parking area) rather than stopping at a random location as they want. The second characteristic is the gradual emergence of the outcome or the development of the intended effect. For example, when the driver steps on the brake pedal, the car usually slows down and then stops, rather than stops abruptly. We examined the effects of these characteristics on SoA in stopping a moving object by manipulating their presence/absence in conditions in which a goal is present or absent (goal-present/absent condition) and a gradual outcome is present or absent (gradual/sudden-stop condition).

When people use automatic control systems, they usually know explicitly that the system functions independently of their control. For example, drivers in autonomous cars know explicitly that the car will move automatically, although they do not control the steering wheel and/or accelerator and a brake pedal. Thus, we examined SoA when instructions informed participants beforehand that the task would include automatic control trials. In addition, as described above, operators sometimes need to exert control over tools even in an automatic control situation, because the system cannot always respond appropriately to specific problems. Our goal is to investigate effective ways to maintain SoA during an automatic control situation to prevent the “out-of-the-loop” performance problem. We intermixed numerous automatic control trials and occasional manual control trials in a single block and compared SoA ratings between these two types of control trials.

## Materials and Methods

### Participants

Twenty-seven young adults (mean age: 21.0 years, 15 women) participated. We did not perform a power calculation because, to our knowledge, no previous studies have examined SoA modulation during automatic control situations. However, this sample size was comparable to those of recent studies on SoA that had 20–30 participants (e.g., Franck et al., 2001; Farrer et al., 2008; Wen et al., 2015b,c). All were naïve to the purpose of this study and had a normal or corrected-to-normal vision. Three participants (one female) were left-handers. This experiment was approved by the Institutional Review Board of Riken and written informed consent was obtained from all participants. The treatment of the participants was conducted in accordance with the Declaration of Helsinki.

### Apparatus, Stimuli, and Tasks

Presentation of stimuli and recording of participant responses were controlled by a computer using Matlab software with the Psychtoolbox (Brainard, 1997; Pelli, 1997; Kleiner et al., 2007). Stimuli were displayed on a 46-inch liquid crystal display (1920 × 1080 pixels, 60 Hz). Participants responded by keypresses on a standard 10-key pad located in front of them. We did not instruct the participants to respond with a specific hand, and they pressed the response key with the hand that was easiest for them to use.

The background display (76.2° × 47.6° of visual angle) was a gray field on which many small white dots were scattered randomly. The dots were intended to make participants feel that there was friction in the display. In the goal-present conditions, the goal, a gray square (150 pixels × 150 pixels, i.e., 7.0° × 7.0°), was presented on the right side of the display. The center of the goal was 400 pixels (i.e., 18.6°) from the right edge of the display. A black circle (diameter 100 pixels, i.e., 4.7°) was the object to be controlled. On each trial of the experimental task, the circle moved rightward from the left side of the display along an invisible, vertically centered line on the display. The circle’s start position was to the left of the left edge of the display, and thus participants could not view the circle at the start of a trial. The circle’s velocity varied from 600 to 880 pixels/s at 20 pixels/s increments (i.e., 15 velocities; 27.6–39.6°/s), in order to reduce task monotony.

**Figure 1:**
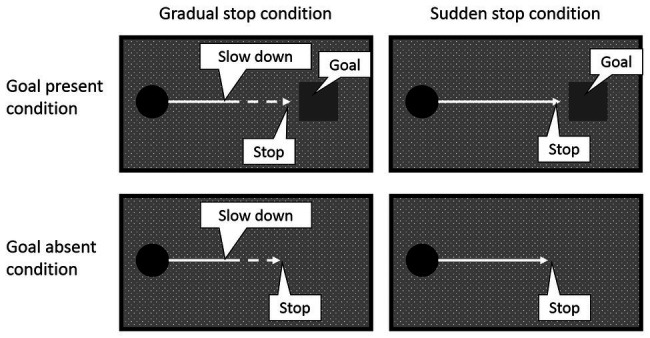
Examples of motion of a circle in four experimental tasks. These four conditions were blocked. Solid lines indicate constant velocity motion, and dashed lines indicate slowing down. On most trials (75% of trials in each block), the circle stopped automatically. Participants were asked to press a key on every trial.

Four experimental conditions were created in a 2 (goal: present/absent) × 2 (type of stop: sudden/gradual) factorial design (see Figure 1). These conditions were blocked, and the block order was randomized between participants. We instructed the participants in all the blocks to press a key once to brake the circle in each trial. We instructed them to aim to stop the circle within the goal square as precisely as possible in the goal-present condition. We told that they could freely stop the circle wherever they wanted in the goal-absent condition. In the sudden-stop conditions, when the brake was applied, the circle stopped abruptly; whereas in the gradual-stop conditions, when the brake was applied, the circle slowed down smoothly with constant deceleration (60 × 9.8 pixels/s^2^, i.e., 27.0°/s^2^) and then stopped. Prior to the experiment, we asked colleagues to observe the circle’s movements (i.e., slowing down), and we confirmed that they felt it was natural.

On each trial, after the circle stopped, a response display appeared. On the response display, participants provided subjective ratings of their SoA by indicating the extent to which they felt that they had stopped the circle by themselves. They typed a number from 1 to 100, with larger numbers indicating a stronger SoA.

### Procedure

Participants sat on a chair positioned 65 cm away from the display and were tested individually. Their head was fixed by a chin rest. After hearing an explanation of the general procedure of the experimental task, they performed 15 practice trials, on which the brake was applied immediately after their keypress, and the circle stopped gradually. The practice trials were conducted in order to familiarize participants with the way the circle stopped and with the response input method (i.e., typing numbers in the response display). Following the practice trials, participants completed four blocks of experimental trials.

Participants were expressly told that every block contained automatic control trials on which the circle would stop independently of their keypress. The location where the circle (i.e., its center) stopped was randomly determined within ±300 pixels (i.e., 14.0°) of the center of the goal on each of the automatic control trials, although the participants were not informed about this. We set an invisible goal in the goal-absent conditions at the same location as the goal-present conditions, and the stop location of the circle was determined the same way as the goal-present conditions. Some catch trials on which the circle stopped by an actual keypress (i.e., manual control trials) were mixed in the block. Manual control trials were included in each experimental block for two reasons. First, our purpose was to compare SoAs during the manual vs. automatic controls. Second, we wanted participants to press a key on every trial because they would feel no SoA without the action (keypress). Actually, in 60 trials in each block, 75% of the trials (45 trials) were automatic control trials and the remaining 25% of the trials (15 trials) were manual control trials. On the manual control trials, the brake was applied 100 ms after the keypress. After the circle stopped, the response display appeared and participants provided subjective ratings of their SoA. In addition to recording the SoA rating in each trial, we also recorded the participants’ keypressing time (elapsed time from trial onset to participant keypress), the brake applied time (elapsed time from trial onset to brake application onset), the stop time of the circle (elapsed time from trial onset to circle stop), and the stop location of the circle. The next trials started 500 ms after the participants provided the rating in the response display.

Even if participants did not press the key to stop the circle, the trial terminated about 1,000 ms after the circle passed through the right edge of the display (i.e., the circle could not be seen in the display) on the manual control trials or 1,000 ms after the circle stopped on the automatic control trials. In these cases, a warning display was presented to remind them to press the key, and then the response display appeared.

## Results and Discussion

We omitted from analysis trials on which participants failed to perform the task because they did not press the key to stop the circle. These were 2.0% of the trials.

### Decrement in SoA by Automatic Control

**Figure 2:**
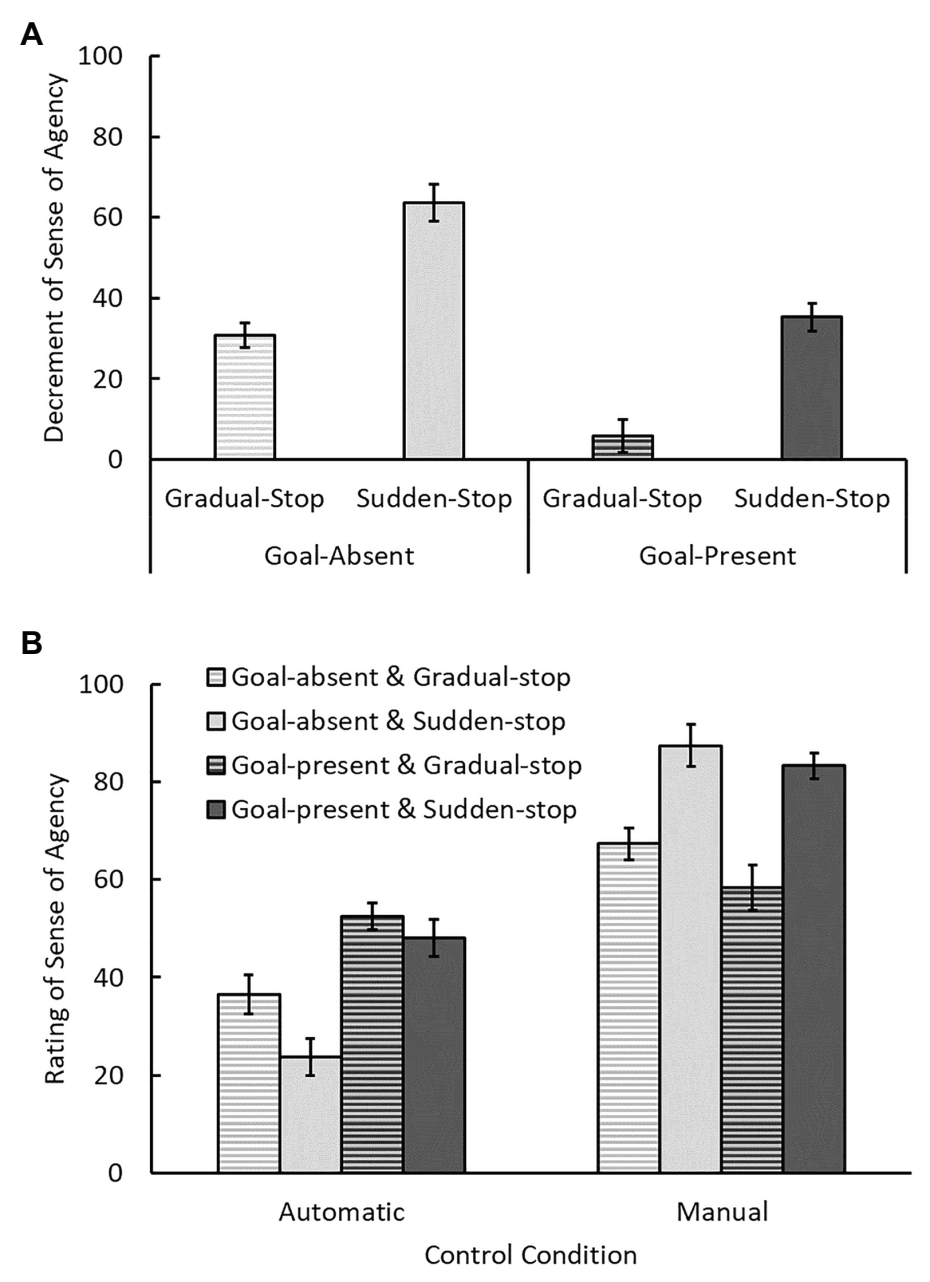
Results of the experiment. **(A)** Sense of agency (SoA) decrements: differences in SoA between manual and automatic control trials. **(B)** Subjective ratings of SoA on manual and automatic control trials. Error bars indicate 95% confidence intervals.

We compared the decrements in SoA caused by automatic control in each condition (Figure 2A). This decrement was defined as the difference in the ratings of manual and automatic control trials (i.e., the mean rating on the manual control trials minus that on the automatic control trials). The positive decrement values found in all conditions suggest that overall ratings were higher on the manual control trials than on the automatic control trials. This was the expected result because participants did control the circle by themselves on the manual control trials, whereas they did not do so on the automatic control trials (Berberian et al., 2012a). An ANOVA on decrements with within-participants factors of type of stop and goal revealed that decrements were significantly smaller in the gradual-stop condition than in the sudden-stop condition, *F*(1, 26) = 142.59.13, *p* < 0.001, *η_p_*^2^ = 0.85, and also smaller in the goal-present condition than in the goal-absent condition, *F*(1, 26) = 68.30, *p* < 0.001, *η_p_*^2^ = 0.72. The interaction was not significant, *F*(1, 26) = 0.80, *p* = 0.38, *η_p_*^2^ = 0.03. It is noted that the decrement value had a significant trend to be larger than 0 even in the goal-present and gradual-stop conditions, *t*(26) = 1.92, *p* = 0.067, and the values in other conditions were significantly larger than 0, *t*s > 9.62, *p*s < 0.001.

These results indicate that the combination of goal-directed action and the gradual emergence of the outcome, i.e., tool-use characteristics, attenuated the decrement in SoA resulting from automation, although the decrement was not wholly eliminated. Therefore, these tool-use characteristics could be effective for maintaining SoA when automatically controlling an object.

### Effects of Gradual-Stop and Goal on SoA Ratings in Automatic and Manual Control Trials

We showed that the two characteristics (i.e., goal-directed action and the gradual emergence of the outcome) attenuate the decrement in SoA by the automation. Next, we examined how these characteristics influence SoA ratings to investigate why they attenuate the SoA decrement by the automation. SoA ratings on automatic and manual control trials were compared (Figure 2B), by means of ANOVA with within-participants factors of control type (automatic/manual), goal, and type of stop. Our main interest in this analysis was examining whether the effects of goal and type of stop varied with control type. Therefore, we focus on the interactions between each of these factors and control type. Before the main results, we confirmed that, as expected, the main effect of control type was significant, *F*(1, 26) = 193.50, *p* < 0.001, *η_p_*^2^ = 0.88, with higher SoA ratings on the manual control trials than on the automatic control trials.

The interaction between control type and type of stop was significant, *F*(1, 26) = 142.59, *p* < 0.001, *η_p_*^2^ = 0.85, with higher ratings in the gradual-stop condition than in the sudden-stop condition on the automatic control trials, *p* < 0.001, but lower ratings in the gradual-stop condition on the manual control trials, *p* < 0.001. The interaction between control type and goal was also significant, *F*(1, 26) = 68.30, *p* < 0.001, *η_p_*^2^ = 0.72, with higher ratings in the goal-present condition than in the goal-absent condition on the automatic control trials, *p* < 0.001, but lower ratings in the goal-present condition on the manual control trials, *p* = 0.025. Although the main effects of goal, type of stop, and the interaction of them were significant, *F*(1, 26) = 13.47, *p* = 0.001, *η_p_*^2^ = 0.34; *F*(1, 26) = 7.76, *p* = 0.010, *η_p_*^2^ = 0.23; *F*(1, 26) = 5.54, *p* = 0.026, *η_p_*^2^ = 0.18, respectively, there were no differences relevant to the present investigation. The three-way interaction was not significant, *F*(1, 26) = 0.80, *p* = 0.38, *η_p_*^2^ = 0.03.

The interaction between control type and type of stop indicated that the gradual emergence of the outcome decreased SoA in manual control trials, but increased SoA in automatic control trials. When the circle stopped abruptly, it was relatively easy to judge whether or not manual braking was applied, because the timing of outcome emergence was clear. This may have produced the very low ratings for automatic control trials and the high ratings for manual control trials in the sudden-stop condition. In contrast, when the circle slowed down and stopped (i.e., in the gradual-stop condition), the time at which the brake was applied was unclear, making it difficult to judge whether it was controlled automatically or manually accurately. Consequently, the SoA ratings on manual and automatic control trials became similar in the gradual-stop condition. That is, the ratings became lower for manual control trials but higher for automatic control trials.

Spatiotemporal inconsistency between the intended action and its outcome has been shown to reduce SoA (e.g., Sato and Yasuda, 2005; Farrer et al., 2013; Kawabe, 2013; Kawabe et al., 2013; Wen et al., 2015a). In the gradual-stop condition, the perceived longer delay from the onset of the action (the keypress) to the occurrence of the outcome should have increased temporal inconsistency, and the difference between the way of action (pressing a key once) and the way of stopping, (slowing down gradually) should also increase the action-outcome inconsistency. However, the SoA ratings in the gradual-stop condition were greater than in the sudden-stop condition on the automatic control trials. Thus, spatiotemporal inconsistency itself in the gradual-stop condition cannot explain the present results fully. This implies that higher cognitive processes, such as inference or interpretation, can be involved in determining SoA during the automatic control situation.

The interaction between control type and goal indicates that the presence of a goal decreased SoA in manual control trials, but increased SoA in automatic control trials. There are two possible reasons why the presence of a goal impaired SoA in manual control trials. Firstly, a goal might impose a constraint on the observer’s action and this attenuates the feeling of “doing by myself.” SoA decreases if the action is restricted by external forces rather than by the manipulator’s free will (Tsakiris et al., 2006; Wenke et al., 2010; Barlas and Obhi, 2013). The second reason is related to the suggestion that SoA ratings might change postdictively because of performance-based agency modulation. Previous studies (e.g., Wen et al., 2015a; Inoue et al., 2017) have reported that SoA increases with improved task performance induced by noticed or unnoticed assistance, suggesting that people might misattribute good performance of control to their own actions. Conversely, ideal outcomes and performances are rarely achieved in regular tool-using situations without assistance. If performance feedback induced by the presence of a goal does not meet expectations, it would probably impair SoA (Wen et al., 2015c).

In contrast, the presence of a goal increased SoA in automatic control trials, possibly due to other mechanisms underlying the above-described process. One possible reason for this increase is that the goal to be achieved is visible, and the automatic control system was programmed to achieve this goal and the intention of observers might accidentally correspond with the outcome produced by the system. As a result, the observers might experience the illusion that the outcome is related to their intention, leading to increased SoA. In other words, the presence of a goal might make observers feel as if the system shares its control with them, i.e., the apparent purpose-sharing between observers and systems. In sum, these effects of having a goal might have resulted in the close ratings between automatic and manual control trials.

### Follow-Up Analysis 1: Effect of Apparent Performance on SoA on the Automatic Control Situation

**Figure 3:**
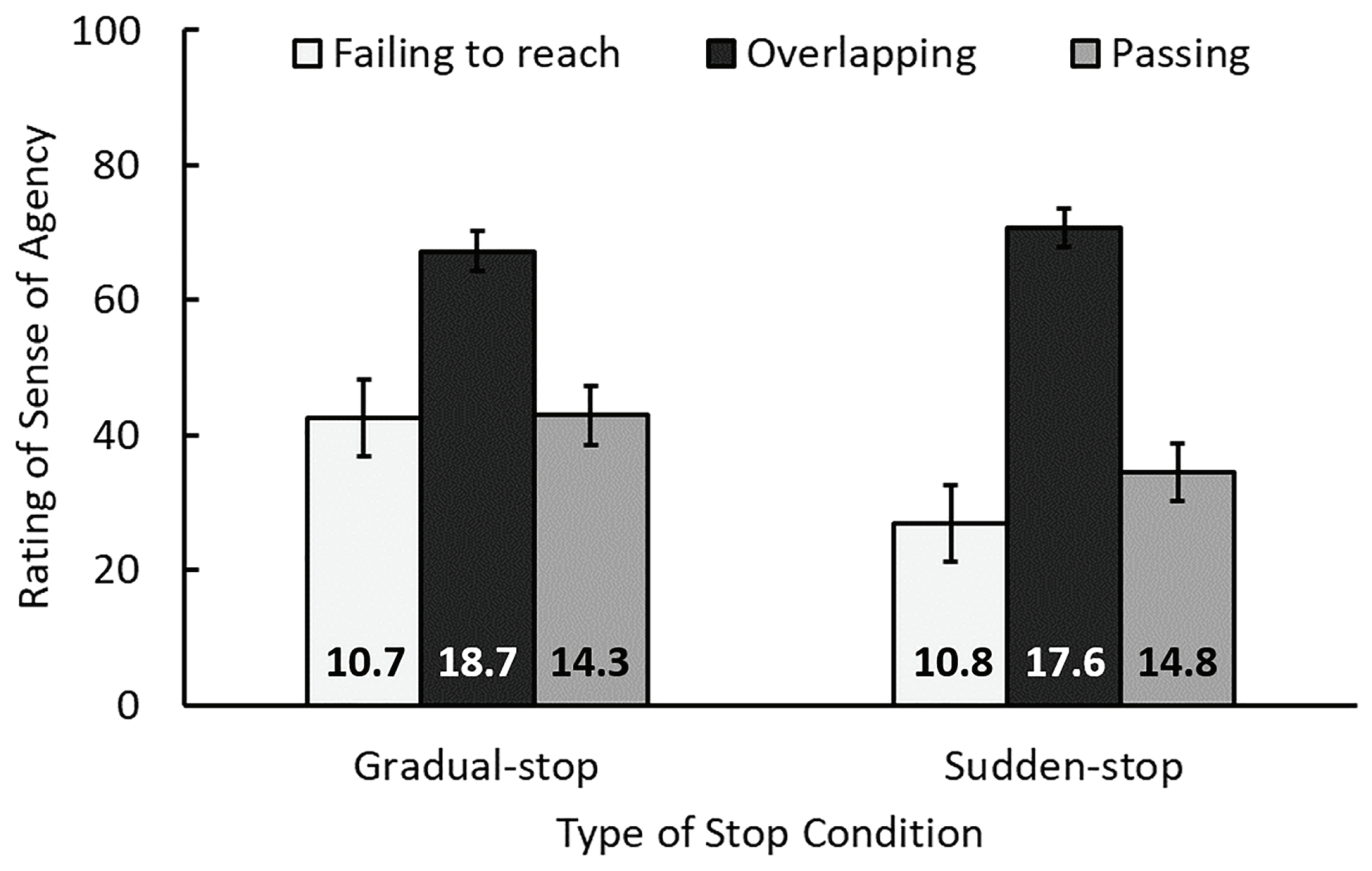
SoA ratings and the number of trials (at the base of each bar) as a function of type of stop and circle stop position. Error bars indicate 95% confidence intervals. Failing to reach: circle stopped to the left of the goal. Overlapping: circle and goal overlapped by at least one pixel. Passing: circle stopped to the right of the goal. The total number of trials for the three stop position conditions is not 45, because approximately one trial of each participant’s data on average was omitted from analysis.

In addition to the main analysis, we conducted some exploratory analyses. We investigated whether the task-performance-based agency modulation (e.g., Wen et al., 2015a; Inoue et al., 2017) is applied to the (illusory) SoA during automatic control situation. We examined the effect of apparent performance on SoA on the automatic control trials. We divided the trials into three groups based on the location at which the circle stopped: failing to reach the goal, overlapping with the goal, and passing the goal (Figure 3). Failing to reach was defined as the circle stopping to the left of the goal (i.e., not overlapping, such that the distance between the centers of the circle and the goal was larger than 125 pixels, i.e., 5.8°). Overlapping was defined as the circle and the goal overlapping by at least 1 pixel. Passing was defined as the circle stopping to the right of the goal (not overlapping). An ANOVA with the factors of type of stop and stop location was conducted. A significant main effect of stop location, *F*(2, 52) = 55.37, *p* < 0.001, *η_p_*^2^ = 0.68, indicated that SoA ratings were higher when the circle overlapped with the goal than when it did not, *p*s < 0.001. This suggests that participants tended to feel that they controlled the object by themselves if task performance was good. The main effect of type of stop, and importantly the interaction between stop location and type of stop were significant, *F*(1, 26) = 8.21, *p* = 0.008, *η_p_*^2^ = 0.24; *F*(2, 52) = 7.42, *p* = 0.002, *η_p_*^2^ = 0.22, respectively. This indicated that when the circle overlapped with the goal, SoAs were comparable for the two types of stop conditions, *p* = 0.18, and that when the circle did not overlap, SoAs were higher in the gradual-stop condition, *p*s < 0.015. This means that SoA decreased drastically when the circle did not overlap with the goal in the sudden-stop condition. In summary, good task performance by the automatic control system may have made people feel postdictively that they controlled the object by themselves.

### Follow-Up Analysis 2: Modulation of Participants’ Keypress Action by the Presence of Goal in the Automatic Control Situation

While the observers felt that the system shares the purpose with them, their actions themselves may be changed by the presence of a visible goal, i.e., they should tend to act appropriately to achieve their purpose in tandem with the automatic control system, rather than acting freely. In order to examine whether the participants’ actions really changed due to the presence of the goal, we compared their keypress timings between the goal conditions (Table 1). An ANOVA revealed that keypress timing was later in the goal-present condition, *F*(1, 26) = 129.53, *p* < 0.001, *η_p_*^2^ = 0.83. As a result, the delay between action and outcome became shorter in the goal-present condition. Keypress timing was earlier in the gradual-stop condition, *F*(1, 26) = 25.22, *p* < 0.001, *η_p_*^2^ = 0.49, because of the longer duration between the application of the brake and the complete stop in the gradual-stop condition. Importantly, the interaction between goal and type of stop was significant, *F*(1, 26) = 19.17, *p* < 0.001, *η_p_*^2^ = 0.42. This interaction indicates that in the goal-present condition, the timing was earlier in the gradual-stop condition, *p* < 0.001, whereas in the goal-absent condition, the keypress timings did not vary with the type of stop, *p* = 0.44. In summary, observers tended to act appropriately when the goal was visible. It should be noted that the keypress timings did not differ between the manual and automatic control trials, *F*(1, 26) = 0.29, *p* = 0.59, *η_p_*^2^ = 0.01, confirming that participants did not know beforehand whether the trials they performed were manual or automatic.

**Table 1:** Mean elapsed times from trial onset to participant keypresses.

	Goal-absent		Goal-present	
	Gradual-stop	Sudden-stop	Gradual-stop	Sudden-stop
Automatic control trials	1.01 (0.06)	1.06 (0.07)	1.62 (0.04)	2.02 (0.04)
Manual control trials	1.02 (0.06)	1.08 (0.08)	1.60 (0.03)	1.99 (0.04)

Mean (SE). The unit is s.

On some automatic control trials, participants pressed the key *after* the circle was slowed down by the automatic system. Although this was not our main interest, we compared SoA ratings between trials, where keypresses occurred before and after the application of the brake. The number of trials with keypresses after the slow-down was larger in the goal-present condition (22.1 and 10.6 trials in the gradual- and sudden-stop conditions, respectively) than in the goal-absent condition (6.4 and 0.6 trials in the gradual- and sudden-stop conditions, respectively), *F*(1, 26) = 134.20, *p* < 0.001, *η_p_*^2^ = 0.84, and also larger in the gradual-stop condition than in the sudden-stop condition, *F*(1, 26) = 55.24, *p* < 0.001, *η_p_*^2^ = 0.68. In the goal-absent conditions, participants pressed the key before the brake application on most of the trials. In the goal-present conditions, we compared SoAs when participants pressed the key before vs. after the brake was applied (Table 2; the data of one participant who never pressed the key after the slow-down in the sudden-stop condition were omitted). An ANOVA revealed that the interaction between type of stop and keypress timing was significant, *F*(1, 25) = 56.75, *p* < 0.001, *η_p_*^2^ = 0.69. SoA ratings decreased when participants pressed the key after the circle slowed down (i.e., stopped) in the sudden-stop condition, *p* < 0.001. In contrast, SoA ratings did not differ in the gradual-stop condition, *p* = 0.15. Participants did not know the precise timing of the brake application, and they tended to attribute the outcome to their actions.

**Table 2:** Mean SoA ratings as a function of keypress timing (before and after brake application) and type of stop on automatic control trials in the goal-present condition.

	Before brake application	After brake application
Gradual-stop	49.1 (3.3)	54.5 (2.8)
Sudden-stop	55.3 (2.7)	23.9 (4.1)

Mean (SE). Data of one participant were removed.

Interestingly, even when participants pressed the key after the circle stopped abruptly in the sudden-stop condition, they may have felt that they controlled the circle. On trials when the keypress occurred after the circle stopped, the lag was only about 150 ms. Participants may have perceived that their keypresses and the circle stop occurred simultaneously, and thus they may have evaluated their agency for stopping the circle postdictively. It should be noted that the range of SoAs was very large across participants, from 1 (no SoA) to 87.5 (strong SoA). Individual differences in sensitivity of time perception and/or degree of self-attribution for the outcome may have affected SoAs. This remains an exciting possibility to examine in future research.

## General Discussion

This study focused on two characteristics of tool-use or object control, goal-directed action and the gradual emergence of the outcome, and examined the effect of these characteristics on the SoA in an automatic control situation. The results indicate that the gradual emergence of the outcome makes it difficult to recognize the timing of the outcome emergence, such that the outcome may be attributed to the observer’s action. Furthermore, the presence of a goal could make the outcome that the observers intend to produce become similar to the actual outcome which the system produces, possibly causing them to modify their action appropriately. As a result, they illusorily attribute the outcome to their own intended action, leading to the feeling of apparently shared purpose between the system and the observer. In addition, observers can estimate their task performance when there is a goal. The outcome usually induces relatively good task performance if the system is programmed to achieve the goal. Good task performance can make them feel that they control the object, and possibly attribute the outcome to their own action. Therefore, the characteristics of goal-directed action and the gradual emergence of the outcome could be effective in maintaining illusory SoA in an automatic control situation.

An illusory SoA is produced as an observer falsely binds an action to its effect in an automatic control situation. Wegner’s theory of “apparent mental causation” (Wegner and Wheatley, 1999; Wegner, 2003) suggests that the experience of willing (or agency) can arise with the conditions of priority, consistency, and exclusivity: Priority indicates that action occurs before the effect, consistency indicates that the action and effect are consistent, and exclusivity indicates that the action is the most plausible cause of the effect. In the automatic control situation, these conditions are not actually satisfied. However, when the effect appears gradually, even though the effect occurs before the action, observers cannot often recognize it, and as a result, priority may be illusorily satisfied. When the goal is visible, the observers develop the intention to accomplish the goal by themselves and act appropriately to accomplish the goal; in other words, consistency and exclusivity may be illusorily satisfied. Thus, observers may tend to feel a strong but illusory SoA. The illusory SoA in the automatic control situation may be generated by a reconstruction process based on inference or interpretation (cf. Haggard, 2005; Metcalfe and Greene, 2007; Wen et al., 2015a).

It is important to keep in mind that maintaining SoA in an automatic control situation is illusorily accomplished. In this study, we intermixed manual control trials and automatic control trials in a single block. The mixture of trials may have been necessary for observers to attribute the control outcome to their actions because they did not know absolutely that the system always controlled the object. If observers felt that they might have sometimes controlled the object by themselves, there would have been an opportunity to bind their action and the outcome. This ambiguity could be material to the illusory maintenance of SoA.

The present results are relevant to SoA in autonomous car driving, in which drivers usually leave driving control to the system but sometimes control it by themselves. According to the structural mechanisms of the vehicle, the outcome of the manipulation (acceleration, deceleration, turning, etc.) usually appears gradually. Thus, a vehicle driving itself has the potential to maintain SoA during the automatic control situation, unless the driver completely engages in other tasks (e.g., reading books, playing games, and sleeping) rather than in driving tasks (e.g., grasping a handle and stepping on pedals). Moreover, the automation system’s outcome, which appeared to match the driver’s intention, may cause the apparent sharing of purpose between the system and the driver. This sharing of purpose is critical for maintaining SoA. Of course, it is impossible to predict the driver’s intentions in every driving situation. However, the driver’s intention to control the situation can be understood in limited driving situations, such as driving ahead in a straight line, turning left or right, or braking, among others. The probability that the driver’s intention matches the outcome accomplished by the system increases in specific driving situations. When drivers explicitly know the automation system’s functions, the apparent match between their intentions and actual outcomes can make them feel that the system achieves the goals that they expect to achieve. They might feel a strong SoA even though they know that they do not control the car. It might be necessary for the automatic system to achieve a goal completely. For example, when an autonomous car stops exactly at a stop line, the driver may feel comfort and strong SoA. We believe that these are important clues for improving the autonomous driving systems. Further research is necessary to clarify the effectiveness of these factors.

The “out-of-the-loop” performance problem implies that manipulators feel they are not using the tool (or the system) at all, and as a result, they are not involved in the tool manipulation. Maintaining SoA could be useful for avoiding this problem. Recognizing the goal (or purpose) is especially important because an intention to manipulate a tool (i.e., to achieve a goal) provides the seeds of SoA. If manipulators have a goal for using a tool and act to achieve this goal, and the outcome produced by the tool (the system) matches this goal, then manipulators would tend to feel that the effect (outcome) of using the tool was the result of their own action, even though the tool (the system) actually produced the outcome. We speculate that manipulators may feel an illusory SoA without having taken an actual action if the outcome is entirely the same as their intention. If the manipulators strongly believe that the system understands their intention (purpose) and controls the object in their place, they should feel an illusory SoA for controlling the object. The belief that the system acts on the manipulator’s behalf may be one of the origins of the illusory SoA in the automatic control situation. We further speculate that the “out-of-the-loop” performance problem can be resolved even if the manipulators only intend and do not actually manipulate the tool, which is a new issue to examine further.

Although our results suggest that the presence of the goal and the gradual emergence of the outcome (i.e., the gradual stopping of the object) increased the subjective rating of SoA for braking the object, there are some limitations. Firstly, it is necessary to keep in mind that this study measured SoA for one-shot object control. This method requires participants’ responses and thus involves continual interruptions of actions (see Wen et al., 2019). To fully understand SoA during an automatic control situation, it is necessary to examine SoA during a continuous control situation (e.g., driving situation). Nevertheless, the present research has provided some suggestions for examining this issue.

Secondly, other factors, such as expertise and fatigue, can influence SoA. Manipulators who have been using automatic control tools for years may feel SoA differently from those who do not use them. Also, when manipulators become tired, they might not want to operate the tool by themselves. The effect of tool-use characteristics on SoA can vary in such cases. Moreover, it is essential to examine SoA for generating an event (e.g., acceleration of an object) as well as SoA for terminating an event (e.g., deceleration of the object) in the case of automatic driving systems. A recent study reported that explicit SoA for generating an event is stronger than SoA for terminating the event (Asai et al., 2019). Therefore, it is important to examine SoA other control types to understand SoA in automatic control fully.

Thirdly, the type of SoA measurement is also important. Although this study focused on explicit aspect of SoA, SoA involves explicit and implicit aspects. One of the most representative implicit agency measurements is the intentional binding effect (IB; Haggard et al., 2002), referring to the fact that observers perceive the duration between their action and its outcome to be shorter when they feel SoA than when they do not. The SoA ratings and IB may reflect different processes (e.g., Synofzik et al., 2013; Wen et al., 2015b). However, they could be related to some degree, at least in simple motor tasks (Imaizumi and Tanno, 2019). Therefore, it is important to investigate IB during automatic control situation.

This study investigated the possible ways of maintaining SoA during automatic control situations to prevent the “out-of-the-loop” performance problem, which is a positive aspect of maintaining SoA in automatic control situations. On the other hand, there is a negative aspect to the illusion of maintaining SoA. For example, illusory SoA without actual control might induce careless control behaviors leading accidents. It is necessary to focus on positive and negative aspects when comprehensively discussing SoA in automatic control situations. Moreover, when considering automation technology in complex situations, it is also important to discuss ethical issues such as personal responsibility for adverse outcomes produced by the system when the person feels the illusory SoA. Many issues remain to be investigated in this field. We believe that results in this study provide preliminary data on the relevance of SoA in automation technology (cf. Wen et al., 2019).

In conclusion, the two characteristics of tool-use or object control focused on in this study, goal-directed action and the gradual emergence of the outcome, could be effective for maintaining illusory SoA even during an automatic control situation. Maintenance of SoA may be necessary to avoid the “out-of-the-loop” performance problem. Although additional research is necessary, our findings should be applicable in developing improved automatic control systems and provide cues for discussing automation technology.

## Data Availability Statement

All datasets presented in this study are included in the article/Supplementary Material.

## Ethics Statement

The studies involving human participants were reviewed and approved by the institutional review board of Riken [Wako3 24-2(8)]. The participants provided their written informed consent to participate in this study.

## Author Contributions

RN developed the study concept, collected and analyzed the experimental data, and drafted the manuscript. TK helped to develop the study concept and drafting the manuscript. All authors contributed to the article and approved the submitted version.

### Conflict of Interest

The authors declare that the research was conducted in the absence of any commercial or financial relationships that could be construed as a potential conflict of interest.
